# 4-Meth­oxy-3-nitro­biphen­yl

**DOI:** 10.1107/S1600536811052846

**Published:** 2011-12-14

**Authors:** Xuqiang Chao, Xiuqin Zhang, Kai Wang, Jun Ji, Qiang Chen

**Affiliations:** a School of Petrochemical Engineering, Changzhou University, Changzhou 213164, Jiangsu, People’s Republic of China; b High Technology Research Institute of Nanjing University, Changzhou 213162, Jiangsu, People’s Republic of China

## Abstract

In the title compound, C_13_H_11_NO_3_, the dihedral angle between the two benzene rings is 36.69 (2)° and the nitro and methy­oxy groups are oriented at 29.12 (14) and 2.14 (12)° with respect to the benzene ring to which they are bonded.

## Related literature

For background information and the synthetic procedure, see: Pourali & Fatemi (2010 ▶). For the crystal structure of a similar compound, see: Marques *et al.* (2008 ▶).
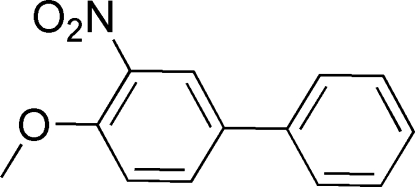


## Experimental

### 

#### Crystal data

C_13_H_11_NO_3_
*M* *_r_* = 229.23
Orthorhombic, 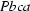

*a* = 7.2464 (14) Å
*b* = 14.416 (3) Å
*c* = 21.270 (4) Å
*V* = 2221.9 (7) Å^3^
*Z* = 8
Mo *K*α radiation
μ = 0.10 mm^−1^
*T* = 296 K
0.20 × 0.18 × 0.15 mm

#### Data collection

Enraf–Nonius CAD-4 diffractometer
Absorption correction: ψ scan (North *et al.*, 1968 ▶) *T*_min_ = 0.981, *T*_max_ = 0.985
23696 measured reflections
2067 independent reflections
1767 reflections with *I* > 2σ(*I*)
*R*_int_ = 0.042
3 standard reflections every 200 reflections  intensity decay: 1%

#### Refinement

*R*[*F* ^2^ > 2σ(*F* ^2^)] = 0.047
*wR*(*F* ^2^) = 0.134
*S* = 1.00
2067 reflections
155 parameters
H-atom parameters constrained
Δρ_max_ = 0.30 e Å^−3^
Δρ_min_ = −0.21 e Å^−3^

### 

Data collection: *CAD-4 Software* (Enraf–Nonius, 1985 ▶); cell refinement: *CAD-4 Software*; data reduction: *XCAD4* (Harms & Wocadlo, 1995 ▶); program(s) used to solve structure: *SHELXS97* (Sheldrick, 2008 ▶); program(s) used to refine structure: *SHELXL97* (Sheldrick, 2008 ▶); molecular graphics: *SHELXTL* (Sheldrick, 2008 ▶); software used to prepare material for publication: *SHELXTL*.

## Supplementary Material

Crystal structure: contains datablock(s) I, global. DOI: 10.1107/S1600536811052846/pv2490sup1.cif

Structure factors: contains datablock(s) I. DOI: 10.1107/S1600536811052846/pv2490Isup2.hkl

Supplementary material file. DOI: 10.1107/S1600536811052846/pv2490Isup3.cml

Additional supplementary materials:  crystallographic information; 3D view; checkCIF report

## Figures and Tables

**Fig. 1.**
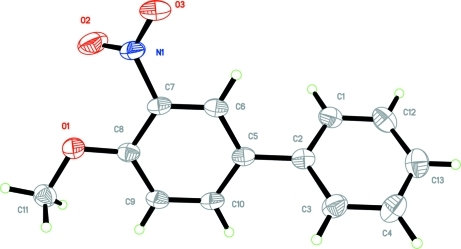
The molecular structure of the title molecule; displacement ellipsoids are drawn at the 50% probability level.

## supplementary crystallographic information

### Comment

The tittle compound is used as an important intermediate in the synthesis of bifenazate which is recognized as an effective miticide (Pourali & Fatemi, 2010).

The bond lengths and angles in the title compound (Fig. 1) are similar to the corresponding bond lengths and angles reported for a closely related compound (Marques *et al.*, 2008). In the title molecule, the torsion angle between the two benzene rings is 36.69 (2)° and the nitro (N1/O2/O3) and methyoxy (O1/C11) groups are oriented at 29.12 (14) and 2.14 (12)°, respectively, with respect to the benzene ring (C5–C10). The crystal structure is devoid of any intramolecular or intermolecular hydrogen bonds.

### Experimental

The title compound was prepared by a method reported in the literature (Pourali & Fatemi, 2010). A solution of 3-nitrobiphenyl-4-ol (2 g, 9.3 mmol) in acetone (20 ml) was added slowly to a solution of dimethyl sulfate (1.2 g, 18 mmol) in an ice bath. After stirring for 48 h at room tempeature, the solvent was evaporated on a rotary evaporator to yield the title compound. Colorless block of the title compound were grown in ethanol by slow slow evaporation of the solvent at room temperature.

### Refinement

The H atoms were positioned geometrically and constrained to ride on their parent atoms, with C—H = 0.93 and 0.97 Å for aryl and methyl H atoms, respectively, with *U*_iso_(H) = *xU*_eq_(C), where *x* = 1.2 for aryl and *x* = 1.5 for methyl H-atoms.

### Crystal data

**Table:**

C_13_H_11_NO_3_	*F*(000) = 960
*M**_r_* = 229.23	*D*_x_ = 1.371 Mg m^−^^3^
Orthorhombic, *P**b**c**a*	Mo *K*α radiation, λ = 0.71073 Å
Hall symbol: -P 2ac 2ab	Cell parameters from 7392 reflections
*a* = 7.2464 (14) Å	θ = 2.8–28.6°
*b* = 14.416 (3) Å	µ = 0.10 mm^−^^1^
*c* = 21.270 (4) Å	*T* = 296 K
*V* = 2221.9 (7) Å^3^	Block, colorless
*Z* = 8	0.20 × 0.18 × 0.15 mm

### Data collection

**Table:**

Enraf–Nonius CAD-4 diffractometer	1767 reflections with *I* > 2σ(*I*)
Radiation source: fine-focus sealed tube	*R*_int_ = 0.042
graphite	θ_max_ = 25.5°, θ_min_ = 1.9°
ω/2θ scans	*h* = −8→8
Absorption correction: ψ scan (North *et al.*, 1968)	*k* = −17→17
*T*_min_ = 0.981, *T*_max_ = 0.985	*l* = −25→13
23696 measured reflections	3 standard reflections every 200 reflections
2067 independent reflections	intensity decay: 1%

### Refinement

**Table:**

Refinement on *F*^2^	Primary atom site location: structure-invariant direct methods
Least-squares matrix: full	Secondary atom site location: difference Fourier map
*R*[*F*^2^ > 2σ(*F*^2^)] = 0.047	Hydrogen site location: inferred from neighbouring sites
*wR*(*F*^2^) = 0.134	H-atom parameters constrained
*S* = 1.00	*w* = 1/[σ^2^(*F*_o_^2^) + (0.0647*P*)^2^ + 1.1253*P*] where *P* = (*F*_o_^2^ + 2*F*_c_^2^)/3
2067 reflections	(Δ/σ)_max_ < 0.001
155 parameters	Δρ_max_ = 0.30 e Å^−^^3^
0 restraints	Δρ_min_ = −0.21 e Å^−^^3^

### Special details

**Table:**

Geometry. All e.s.d.'s (except the e.s.d. in the dihedral angle between two l.s. planes) are estimated using the full covariance matrix. The cell e.s.d.'s are taken into account individually in the estimation of e.s.d.'s in distances, angles and torsion angles; correlations between e.s.d.'s in cell parameters are only used when they are defined by crystal symmetry. An approximate (isotropic) treatment of cell e.s.d.'s is used for estimating e.s.d.'s involving l.s. planes.
Refinement. Refinement of *F*^2^ against ALL reflections. The weighted *R*-factor *wR* and goodness of fit *S* are based on *F*^2^, conventional *R*-factors *R* are based on *F*, with *F* set to zero for negative *F*^2^. The threshold expression of *F*^2^ > σ(*F*^2^) is used only for calculating *R*-factors(gt) *etc*. and is not relevant to the choice of reflections for refinement. *R*-factors based on *F*^2^ are statistically about twice as large as those based on *F*, and *R*- factors based on ALL data will be even larger.

### Fractional atomic coordinates and isotropic or equivalent isotropic displacement parameters (Å^2^)

**Table:**

	*x*	*y*	*z*	*U*_iso_*/*U*_eq_	
O1	0.0839 (2)	0.29025 (9)	0.20268 (6)	0.0550 (4)	
C6	0.0647 (2)	0.25993 (12)	0.37180 (9)	0.0440 (4)	
H6	0.0437	0.2118	0.4000	0.053*	
C10	0.1357 (2)	0.41652 (12)	0.35023 (9)	0.0470 (4)	
H10	0.1629	0.4762	0.3640	0.056*	
C5	0.1047 (2)	0.34788 (12)	0.39446 (9)	0.0429 (4)	
N1	0.0116 (2)	0.14832 (10)	0.28966 (8)	0.0510 (4)	
C7	0.0557 (2)	0.24306 (11)	0.30850 (9)	0.0435 (4)	
C8	0.0879 (2)	0.31220 (12)	0.26371 (8)	0.0433 (4)	
C9	0.1278 (3)	0.39972 (12)	0.28690 (9)	0.0469 (4)	
H9	0.1496	0.4481	0.2589	0.056*	
C2	0.1145 (2)	0.36642 (12)	0.46247 (9)	0.0460 (4)	
O3	0.0541 (3)	0.08624 (9)	0.32547 (8)	0.0732 (5)	
C11	0.1229 (3)	0.36171 (14)	0.15871 (9)	0.0558 (5)	
H11A	0.0316	0.4097	0.1623	0.084*	
H11B	0.1208	0.3366	0.1169	0.084*	
H11C	0.2428	0.3872	0.1672	0.084*	
O2	−0.0705 (3)	0.13539 (11)	0.24097 (9)	0.0828 (6)	
C3	0.2432 (3)	0.42742 (15)	0.48667 (10)	0.0614 (6)	
H3	0.3226	0.4583	0.4594	0.074*	
C1	−0.0019 (3)	0.32329 (14)	0.50488 (10)	0.0586 (5)	
H1	−0.0906	0.2820	0.4902	0.070*	
C4	0.2562 (4)	0.44336 (18)	0.55029 (11)	0.0723 (6)	
H4	0.3444	0.4846	0.5654	0.087*	
C12	0.0112 (3)	0.34047 (17)	0.56840 (11)	0.0670 (6)	
H12	−0.0698	0.3114	0.5960	0.080*	
C13	0.1418 (3)	0.39961 (17)	0.59124 (11)	0.0692 (6)	
H13	0.1524	0.4098	0.6343	0.083*	

### Atomic displacement parameters (Å^2^)

**Table:**

	*U*^11^	*U*^22^	*U*^33^	*U*^12^	*U*^13^	*U*^23^
O1	0.0698 (9)	0.0411 (7)	0.0541 (8)	−0.0008 (6)	0.0041 (7)	0.0009 (6)
C6	0.0395 (9)	0.0348 (8)	0.0578 (11)	0.0008 (7)	0.0014 (8)	0.0070 (8)
C10	0.0436 (10)	0.0329 (8)	0.0645 (11)	−0.0037 (7)	0.0048 (8)	−0.0016 (8)
C5	0.0334 (8)	0.0375 (9)	0.0578 (10)	−0.0006 (7)	0.0001 (7)	0.0015 (8)
N1	0.0559 (10)	0.0314 (8)	0.0657 (10)	−0.0010 (7)	0.0037 (8)	0.0006 (7)
C7	0.0377 (9)	0.0298 (8)	0.0628 (11)	0.0008 (7)	0.0002 (8)	0.0000 (7)
C8	0.0387 (9)	0.0344 (8)	0.0569 (11)	0.0036 (7)	0.0040 (8)	0.0029 (7)
C9	0.0465 (10)	0.0355 (9)	0.0587 (11)	−0.0009 (8)	0.0056 (8)	0.0072 (8)
C2	0.0397 (9)	0.0406 (9)	0.0577 (11)	0.0038 (7)	0.0003 (8)	0.0010 (8)
O3	0.1017 (13)	0.0329 (7)	0.0850 (11)	0.0030 (7)	0.0018 (9)	0.0080 (7)
C11	0.0595 (12)	0.0506 (11)	0.0572 (11)	0.0030 (9)	0.0050 (9)	0.0065 (9)
O2	0.1095 (15)	0.0509 (9)	0.0879 (12)	−0.0175 (9)	−0.0282 (11)	−0.0060 (8)
C3	0.0554 (12)	0.0646 (13)	0.0642 (12)	−0.0117 (10)	0.0057 (10)	−0.0110 (10)
C1	0.0580 (12)	0.0539 (11)	0.0638 (12)	−0.0048 (10)	0.0011 (10)	0.0083 (9)
C4	0.0679 (14)	0.0767 (15)	0.0722 (14)	−0.0068 (12)	−0.0060 (12)	−0.0211 (12)
C12	0.0643 (14)	0.0740 (15)	0.0628 (13)	0.0079 (12)	0.0098 (11)	0.0143 (11)
C13	0.0696 (15)	0.0787 (15)	0.0593 (13)	0.0147 (13)	−0.0017 (11)	−0.0035 (11)

### Geometric parameters (Å, °)

**Table:**

O1—C8	1.336 (2)	C2—C1	1.383 (3)
O1—C11	1.420 (2)	C2—C3	1.381 (3)
C6—C7	1.370 (3)	C11—H11A	0.9600
C6—C5	1.387 (2)	C11—H11B	0.9600
C6—H6	0.9300	C11—H11C	0.9600
C10—C9	1.370 (3)	C3—C4	1.376 (3)
C10—C5	1.384 (2)	C3—H3	0.9300
C10—H10	0.9300	C1—C12	1.377 (3)
C5—C2	1.473 (3)	C1—H1	0.9300
N1—O2	1.209 (2)	C4—C13	1.358 (3)
N1—O3	1.215 (2)	C4—H4	0.9300
N1—C7	1.459 (2)	C12—C13	1.363 (4)
C7—C8	1.398 (2)	C12—H12	0.9300
C8—C9	1.385 (3)	C13—H13	0.9300
C9—H9	0.9300		
			
C8—O1—C11	117.63 (15)	C1—C2—C5	121.99 (17)
C7—C6—C5	120.92 (16)	C3—C2—C5	120.93 (17)
C7—C6—H6	119.5	O1—C11—H11A	109.5
C5—C6—H6	119.5	O1—C11—H11B	109.5
C9—C10—C5	122.37 (17)	H11A—C11—H11B	109.5
C9—C10—H10	118.8	O1—C11—H11C	109.5
C5—C10—H10	118.8	H11A—C11—H11C	109.5
C10—C5—C6	116.83 (17)	H11B—C11—H11C	109.5
C10—C5—C2	122.02 (16)	C4—C3—C2	121.3 (2)
C6—C5—C2	121.15 (16)	C4—C3—H3	119.4
O2—N1—O3	123.26 (17)	C2—C3—H3	119.4
O2—N1—C7	119.17 (16)	C12—C1—C2	121.1 (2)
O3—N1—C7	117.51 (17)	C12—C1—H1	119.4
C6—C7—C8	122.36 (16)	C2—C1—H1	119.4
C6—C7—N1	116.53 (16)	C13—C4—C3	120.8 (2)
C8—C7—N1	121.11 (17)	C13—C4—H4	119.6
O1—C8—C9	124.48 (16)	C3—C4—H4	119.6
O1—C8—C7	119.30 (16)	C13—C12—C1	120.7 (2)
C9—C8—C7	116.20 (16)	C13—C12—H12	119.7
C10—C9—C8	121.33 (16)	C1—C12—H12	119.7
C10—C9—H9	119.3	C4—C13—C12	119.1 (2)
C8—C9—H9	119.3	C4—C13—H13	120.5
C1—C2—C3	117.07 (19)	C12—C13—H13	120.5
			
C9—C10—C5—C6	0.0 (3)	C5—C10—C9—C8	0.0 (3)
C9—C10—C5—C2	179.66 (17)	O1—C8—C9—C10	−177.71 (17)
C7—C6—C5—C10	−0.4 (3)	C7—C8—C9—C10	0.3 (3)
C7—C6—C5—C2	179.97 (16)	C10—C5—C2—C1	144.19 (19)
C5—C6—C7—C8	0.7 (3)	C6—C5—C2—C1	−36.2 (3)
C5—C6—C7—N1	−179.67 (15)	C10—C5—C2—C3	−36.6 (3)
O2—N1—C7—C6	149.8 (2)	C6—C5—C2—C3	143.06 (19)
O3—N1—C7—C6	−27.5 (2)	C1—C2—C3—C4	0.9 (3)
O2—N1—C7—C8	−30.6 (3)	C5—C2—C3—C4	−178.4 (2)
O3—N1—C7—C8	152.12 (18)	C3—C2—C1—C12	−0.3 (3)
C11—O1—C8—C9	−0.1 (3)	C5—C2—C1—C12	178.99 (19)
C11—O1—C8—C7	−178.07 (16)	C2—C3—C4—C13	−0.3 (4)
C6—C7—C8—O1	177.44 (16)	C2—C1—C12—C13	−1.0 (3)
N1—C7—C8—O1	−2.1 (2)	C3—C4—C13—C12	−1.0 (4)
C6—C7—C8—C9	−0.7 (3)	C1—C12—C13—C4	1.6 (4)
N1—C7—C8—C9	179.73 (16)		
